# The dementia research career pipeline: Gender disparities in publication authorships and grant funding outcomes at different career stages

**DOI:** 10.12688/amrcopenres.13072.1

**Published:** 2022-08-10

**Authors:** Melina Andreou, Narshil Choi, Jorge Gómez Magenti, Susan Kohlhaas, Rosa Sancho

**Affiliations:** 1 Imperial College London, London, SW7 2BX, UK; 2 Drexel University, Philadelphia, 19104, USA; 3 Alzheimer's Research UK, Cambridge, CB21 6AD, UK

**Keywords:** gender disparities, dementia research, research funding, grant applications, author lists

## Abstract

**Background:**

Multiple studies have analysed gender disparities in academic research. Here we study the gender composition of the dementia research field at different stages in the career pipeline.

**Methods:**

We use various data sources to gain insights about the gender ratio across career stages: conference attendance data as a proxy for the field as a whole; bibliometric data to know who publishes, and who occupies positions of seniority among the listed authors; and Alzheimer’s Research UK’s (ARUK) internal grant funding data to understand who obtains funding. We also analyse the scoring of grant applications based on the gender of the reviewers.

**Results:**

Our results confirm that female researchers leave dementia academic research at higher rates than men, before transitioning into senior positions. In 2020, they comprised over 60% of the field, produced 54% of first authorships, but only accounted for 38% of last authorships. Overall, women received 37% of ARUK’s competitive grants, with significant differences between grant schemes awarded for early career researchers (64% female awardees) compared to grant schemes aimed at senior researchers (33% female awardees). Men and women applied for and obtained grants at significantly different rates depending on the career stage at which the grant was aimed.

Finally, we also observed that male and female reviewers apply evaluation criteria differently, with men giving better scores than women on average.

**Conclusions:**

Our study adds to the evidence that shows that women get published less, receive less funding, and transition into senior academic positions at disproportionally lower rates than men do. We briefly discuss potential reasons why gender disparities arise as researchers progress into senior positions, and offer interventions ARUK can implement in its application and evaluation process to address those disparities.

## Plain Language Summary

Alzheimer’s Research UK has conducted work to identify and address gender disparities along the career pipeline in dementia research.

We used a mix of data from scientific publications (from more than 180,000 dementia research papers published between the years 2000 and 2020) and internal funding data (from more than 2,000 grant applications) to show that female researchers leave academia at higher rates than men before transitioning into senior positions. Women occupy fewer senior positions in authors list, despite being the majority of junior authors; they apply in lower numbers to senior grants schemes, despite being the majority of applicants to early career schemes; and they have lower success rates in obtaining funding for senior grant schemes, despite having higher success rate in junior calls.

Moreover, our analysis shows that while the overall gender gap has been closing, the rate of change at senior positions has been slower than at junior positions, which indicates that women disproportionately face barriers that make their career progression more difficult.

Alzheimer’s Research UK is using this information to inform programmes that will help brilliant researchers succeed in their careers without being hindered by external factors. This will allow us to for people with dementia.

## Introduction

Multiple analyses of publication and funding data have shown that women’s research outputs and rates of career progression in academia are below those of their male colleagues, and below what would be expected based on their representation in their fields of work.

### Discrepancies in authorship ratios

A common way of discerning research output quantity is by counting the number of co-authorships in scientific publications (i.e., how many times the name of a researcher appears in the list of authors of peer-reviewed publications). Due to an unwritten agreement in most scientific disciplines, the seniority and the author’s importance within a publication can be inferred based on the co-authorship position. In this manner, first and last authors are widely considered to be the key positions in the author list, with the first author often being an early career researcher (ECR) and the last author being a senior researcher or the principal investigator of the study.

The gender ratio in co-authorships has been calculated for big datasets in all fields of scholarly research ^1^, in various Science, Technology, Engineering, Mathematics, and Medicine (STEMM) fields ^2, 3^, and in subject or location-specific analyses ^4– 10^. Analyses directly related to this paper’s field of research include one by Shen *et al*. that looks at women’s underrepresentation in high profile journals in the field of neuroscience ^11^, and work by Menzel *et al*. that looks specifically at gender disparities in Alzheimer’s and dementia research ^12^. These studies reach similar conclusions: 1) women tend to have fewer co-authorships than men; 2) women publish less than men (their co-authorships/author ratio is lower); 3) last author positions are disproportionally held by men; and 4) while the overall gender gap has been closing due to the number of women in research having increased, the rate of change at senior positions has been slower, which may indicate that women disproportionately face barriers that make their career progression more difficult.

### Discrepancies in access to funding and career progression

In the UK, 47% of academic staff are female, but they comprise 39% of staff employed on senior academic contracts and only 28% of professors ( https://www.hesa.ac.uk/news/19-01-2021/sb259-higher-education-staff-statistics). In the EU, among all the research positions held by women, 7.4% of them are Grade A (the highest research positions available). At the same time, 16.7% of all positions occupied by men are Grade A. And despite women being 39% of graduates at doctoral level in STEM disciplines, they comprise 35% of grade C, 28% of grade B and 15% of grade A academic staff overall ^13^.

Parallel dynamics can be seen in the allocation of research funding. While women comprise the majority of applicants for the National Institute for Health Research (NIHR) grants at pre-doctoral, doctoral, and post-doctoral stages, this number goes down to 37% at senior investigator level ( https://www.nihr.ac.uk/documents/diversity-data-report-202021/29410). Women receive 36% of competitive awards in Science from the Wellcome Trust, and in 2019/20 they received only 29% of the grants over £1M. This means that men received £250M in scientific grants compared to women’s £115M ( https://wellcome.org/reports/grant-funding-data-2019-2020). The NIHR and Wellcome Trust are examples of the two most prominent government and charity funding bodies in the UK, respectively, but similar disparities are found for other funders ( https://www.cancerresearchuk.org/sites/default/files/cruk_diversity_data_in_our_grant_funding_2017-2019_feb_2021.pdf).

### Potential reasons for these discrepancies

An obvious reason women might produce fewer research outputs and show lower rates of progression in their careers in academia is maternity. Extended leave from work has been shown to have detrimental effects on career progression and wage-earning potential in other fields of work ^14, 15^. Increased paternity leave and other social care policies have been suggested to be effective interventions to diminish motherhood penalties ^16, 17^. However, a multitude of evidence points towards other factors operating on top of maternity leave ^18– 29^.

Canadian researchers used the creation of two new funding schemes with different evaluation criteria as a natural experiment to look at grant applications from principal investigators to the Canadian Institutes of Health Research. The overall grant success rate was 15.8%. After adjusting for age and research domain, they found that when one of the new review processes focussed on the scientific merit of the proposal the gap between men and women was the same as in the older funding scheme: men presented 0.9 percentage points higher success rates (3.2 higher–1.4 lower). However, when the second new review process focussed on the calibre of the principal investigator, the gap was 4.0 percentage points (6.7 higher–1.3 lower) ^18^. This shows that evaluation criteria that are influenced by personal stereotypes or preconceptions can affect the success rates of women applying to senior positions.

Publishing in “high-quality” journals is often a requisite to access funding, especially for grant calls for senior programmes. As mentioned earlier, studies show that women are less likely to be co-authors in articles published in “high-quality” journals. As little as 18.1% of last authorships in prestigious journals are held by women ^19^, and in the field of neuroscience, the percentage of female first and last authors has a significant negative association with increasing journal impact factor ^11^. However, after some journals introduced a double-blind process, in which the reviewer did not know the exact identity of the authors, there was an uptick in the number of publications authored by women that were accepted ^20, 21^.

Citation metrics are often used to determine the quality of past research by reviewers. There is ample evidence that gender plays a significant role in citation patterns. Not only have men been found to self-cite more often than women ^22^, but who gets cited is related to the gender of the first and last authors doing the citing ^23^. Dworkin *et al*. showed that papers with male first and last authors (MM) overcited other MM publications, and under-cited research in which women were involved in positions of authority. There has also been a growth in this discrepancy observed in the past decade, which shows that this type of citing behaviour is not only active but growing. This gender homophily has been found as well in the establishment of scientific collaborations (i.e., men disproportionately collaborate more with men, and women collaborate more with women) ^24^.

Gender has been suggested to play a role in the manuscript peer review process ^25^, in the vocabulary used by reviewers to review grant applications ^26^, in the vocabulary researchers use to talk about their research ^27^, and even in how often men and women ask questions at scientific conferences ^28^. Moreover, whether women ask more or fewer questions at conferences is also related to the gender of the person chairing the sessions, and interventions as simple as calling a female delegate for a first question increases the number of questions asked by other women in the room ^29^.

It is important to note that several aspects discussed above are not independent of each other; gender imbalances in one factor will likely have a negative impact in some of the other metrics.

### Contribution to the field

**Figure 1.**
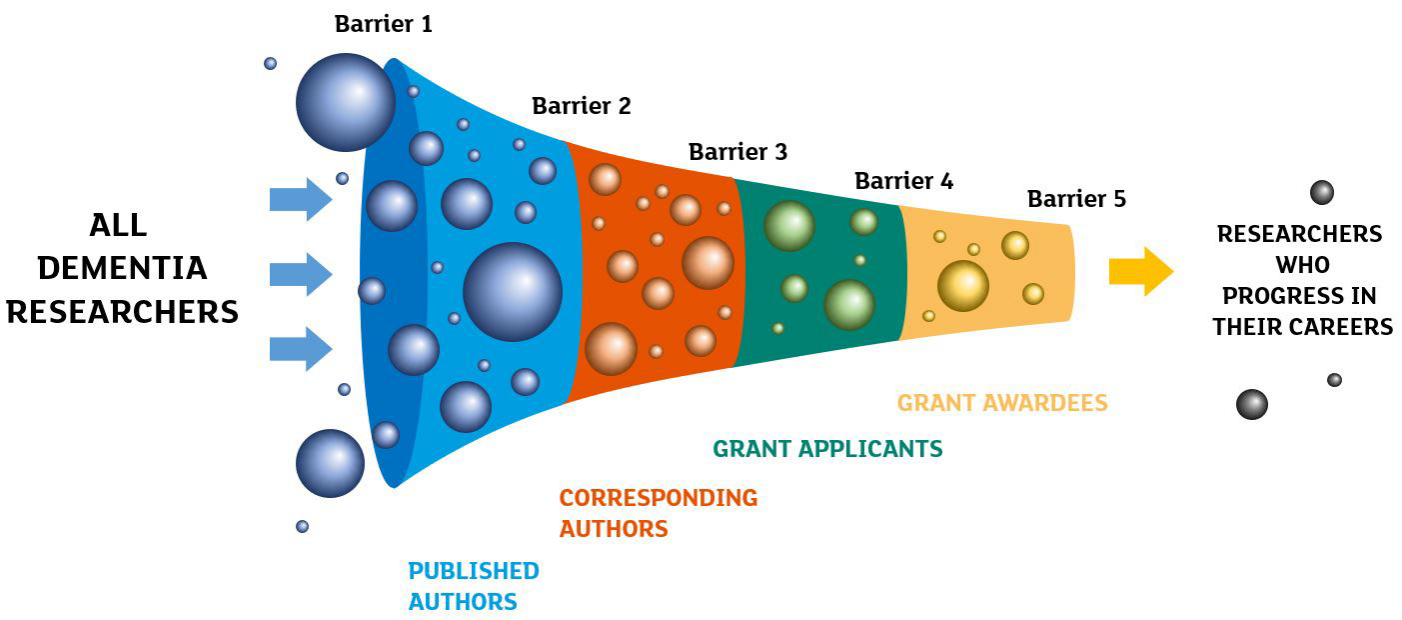
Schematic representation of the filters or barriers researchers need to go through to progress in their academic careers.

This publication is the first to produce a full view of the career pipeline of dementia researchers in academia, based both on bibliometric and funding data ( Figure 1). In the following sections, we analyse gender discrepancies in publication rates, authorship position, application and success rates to grant schemes of a major dementia research charity. We analyse how these metrics change at different career stages. We also investigate whether applicants’ success rates to grant applications are influenced by the gender of the reviewer.

These results are informing a series of programmes at Alzheimer’s Research UK aimed at: 1) reducing potential bias in our grant evaluation and allocation process; 2) improving the current situation of women disproportionately leaving their academic careers before transitioning into senior positions; and 3) ultimately, ensuring we fund the best quality science. These interventions will be briefly discussed at the end of this paper.

## Methods

### Gender analysis

**Inferring gender from authors’ first names:** An online gender API ( www.gender-api.com) was used to computationally determine the gender of researchers’ first names (male or female). While this binary approach does not reflect the reality of gender identification, and ideally each individual author would self-identify, it still proves a powerful tool to analyse at scale datasets of names that can give insights on different trends related to gender. The API provides a label (male, female, unknown) and quantifies the confidence on that assignment. As other similar analyses have done in the past, we only took into account the gender assignments that had a statistical confidence greater or equal to 95%. At the time when we accessed the API to retrieve names and assigned genders (July 2020) this criterion was not commonly met by names of Asian origin, so we decided to focus the analysis on Western countries only.

**Inferring gender from awardees’ pictures:** To double-check the accuracy of the API, we manually searched 468 ARUK grant awardees. We were able to infer the gender of 463 researchers via pictures in academic profiles (via university or departmental webpages) or professional profiles (e.g., LinkedIn).

### Data sources

We use various data sources to gain insights about the gender ratio across career stages: conference attendance data as a proxy for the field as a whole; bibliometric data to know who publishes, and who occupies positions of seniority among the listed authors; and ARUK’s internal grant funding data to understand who obtains funding. We also analysed the scoring of grant applications based on the gender of the reviewers. Internal data and analyses files can be found on https://github.com/jorgoma/gender-analysis-paper ^30^, and raw files with publication data can be found on https://zenodo.org/record/6535739#.YuERfz3MKUk ^31^.

**Conference data:** Conference attendance was obtained from summary data provided by colleagues at the Alzheimer’s Association, on the attendance at the 2019, 2020 and 2021 Alzheimer’s Association International Conference ( Table 1).

**Table 1.** Attendee gender distribution at the Alzheimer’s Association International Conference.

Attendee gender	2019- Los Angeles (In person only)	2020- (Virtual)	2021- Denver (Hybrid)
Female	50%	65%	59%
Male	50%	34%	40%
All other genders	NA	1%	0%

**Publication data:** Dementia research publications metadata were downloaded from Dimensions in October 2021. The terms (dementia OR Alzheimer*) were searched in the title or abstract of publications. These terms were chosen because they showed the best grant coverage for top dementia-research-only funders, among different filtering criteria that were tried ( Table 2). At the same time, these terms restricted the results to only dementia-related publications, unlike broader filtering criteria such as “Neurological”, from the Health Research Classification System (HRCS) ( https://hrcsonline.net/).

**Table 2.** Grant coverage for dementia research funders using different search criteria in Dimensions.

Search criteria		Alzheimer's Research UK	Alzheimer's Society	Alzheimer's Association	Alzheimer's Drug Discovery Foundation	Alzheimer's Society of Canada
HRCS	Neurological	88%	75%	97%	62%	86%
RCDC	Acquired Cognitive Impairment OR Dementia OR Alzheimer's Disease including Alzheimer's Disease Related Dementias (AD/ADRD) OR Alzheimer's Disease OR Alzheimer's Disease Related Dementias (ADRD) OR Frontotemporal Dementia (FTD) OR Parkinson's Disease OR Lewy Body Dementia OR Vascular Cognitive Impairment/Dementia OR Down Syndrome	91%	72%	99%	62%	94%
keywords in (title OR abstract)	dementia OR alzheimer*	95%	90%	99%	78%	96%
mixed	HRCS "Neurological" AND (dementia OR alzheimer*) in (title OR abstract)	85%	73%	96%	60%	85%

We focused our analysis on the top 10 Western countries by the number of publications, due to the limitations in the confidence of gender assignment for names of Asian origin. The countries included in this analysis were: United States, United Kingdom, Germany, Canada, Italy, France, Australia, Spain, Sweden and Netherlands. We restricted the analysis to publications from the year 2000 until the year 2020.

Metadata on 180,158 dementia research publications between 2000 and 2020 were downloaded, cleaned, and analysed in R version 4.0.2. Each contained at least one co-author based in one of the top ten Western countries by production in the field. Authorships, first authorships, last authorships, and unique authors were extracted and analysed by year of publication and country of affiliation. Data was examined using descriptive analysis.

**Grant application data:** Historical records of applications to ARUK grant schemes were used. 2,148 applications to competitive grants were included in the analysis, of which 1,999 (93%) were assigned to a gender. Applicants with an unknown gender were excluded from the analysis. 683 grants were funded or partially funded, of which 652 grants (95%) were assigned to a gender.

**Grant scoring data:** Historical records of reviews of ARUK grant applications were used. There were 1,556 instances of a peer-reviewer scoring a grant, of which 1,384 (89%) were assigned to a gender. There were 8,977 instances of a member of our Grant Review Board providing a score.

### Grant application analysis

Using the ARUK grant application data, the effect of the gender of the applicant on the outcome of the funding application was examined using descriptive analysis, Chi-squared analysis, Mantel-Haenszel, and logistic regression.

The dependent variable, the outcome of the application, was categorized as funded (which included both fully and partially funded grants) and not funded. The analysis was not adjusted for missing data and assumed no differences between those with missing data. Descriptive analysis was used to summarize the outcome variable (funded) for gender and type of project. To control for confounding factors and to assess for potential effect modification, the data were assessed by stratification using Mantel-Haenszel and multivariate regression. The Mantel-Haenszel test examined whether there were statistically significant differences between male and female applicant’s success of the application, while controlling for type of project. Univariate and multivariate analysis was conducted using logistic regression.

## Results

### Conference data

Women comprised between 50% (2019 AAIC- in person only in Los Angeles) and 65% (2020 AAIC- fully virtual) of attendees to major dementia research conferences in the years 2019 to 2021. The 2021 hybrid conference was attended by over 11,000 delegates.

### Bibliometric analysis

There was a total of 236,952 unique authors in this dataset with 153,365 assigned to a gender. Unique authors presented a gender ratio (ratio of male authors to female authors) of 1.15. These authors produced 567,842 authorships, with a gender ratio of 1.5. A total of 89,776 last authorships had a gender ratio of 2.25 ( Table 3).

**Table 3.** Gender distribution of unique authors, authorship, last authors in dementia research publications.

	Total	Male		Female		Gender ratio
Type of authorship	n	n	%	n	%	
Unique authors	153,365	80,924	52.7	72,751	47.3	1.15
Authorships	567,842	339,677	59.8	228,165	40.2	1.5
Last authorship	89,776	62,003	69.1	27,773	30.9	2.25

Another way of thinking about this ratio discrepancy between authorships and authors is in terms of the average number of publications each researcher produces: 3.1 publications co-authored by female researchers and 4.2 co-authored by male researchers, a 35% difference.

We explored what type of researcher was driving this difference in publication output (i.e., ‘productivity’). ‘Productivity’ was categorized by having produced 10 or fewer publications, and more than 10 publications. As shown in Table 4, among those with 10 or fewer publications, the gender ratio in the number of authorships was 1.13 (men produced 13% more authorships). On the other hand, among those who have co-authored more than 10 publications, the gender ratio was 2.01, meaning that men have produced 101% more authorships than female authors.

**Table 4.** Gender distribution of total authorships produced by authors with different levels of ‘productivity’.

	Total authorships	Female		Male		Gender ratio
**‘Productivity’**	n	n	%	n	%
Authors with 10 or fewer articles	288,326	135,185	46.9	153,131	53.1	1.13
Authors with more than 10 articles	279,516	92,980	33.3	186,536	66.7	2.01

**Figure 2.**
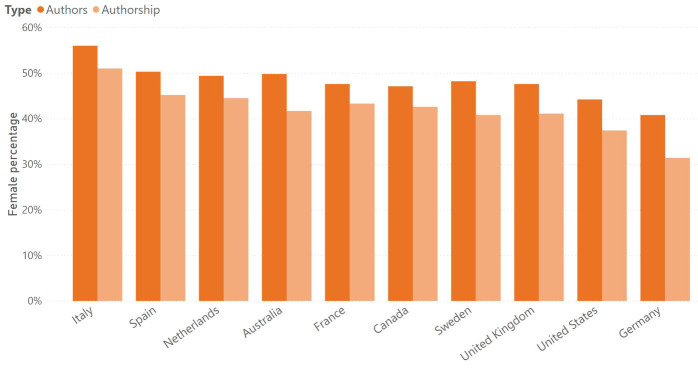
Percentage of female authors and authorships.

It is important to note that these discrepancies between the number of authors and authorships is widespread in all the countries included in this analysis ( Figure 2). For instance, this analysis agrees with previous work that shows low levels of women conducting dementia research in Germany ^2^, where only 41% of authors are female, and females only produce 31% of the authorships.

The past 20 years have seen the gender gap in scientific production and positions of academic authority steadily closing ^2^. Our data suggest that those trends hold for dementia research. In 2020, half of the countries included in this analysis had reached 50% or more of female authors, and only the United States and especially Germany lagged considerably behind the gender parity mark ( Table 5).

**Table 5.** Percentage of female authors in 2020 in the countries included in this analysis.

Country	Percentage of female authors in 2020
Italy	55
Netherlands	51
Spain	51
Australia	50
Canada	50
United Kingdom	48
France	48
Sweden	48
United States	45
Germany	40

**Figure 3.**
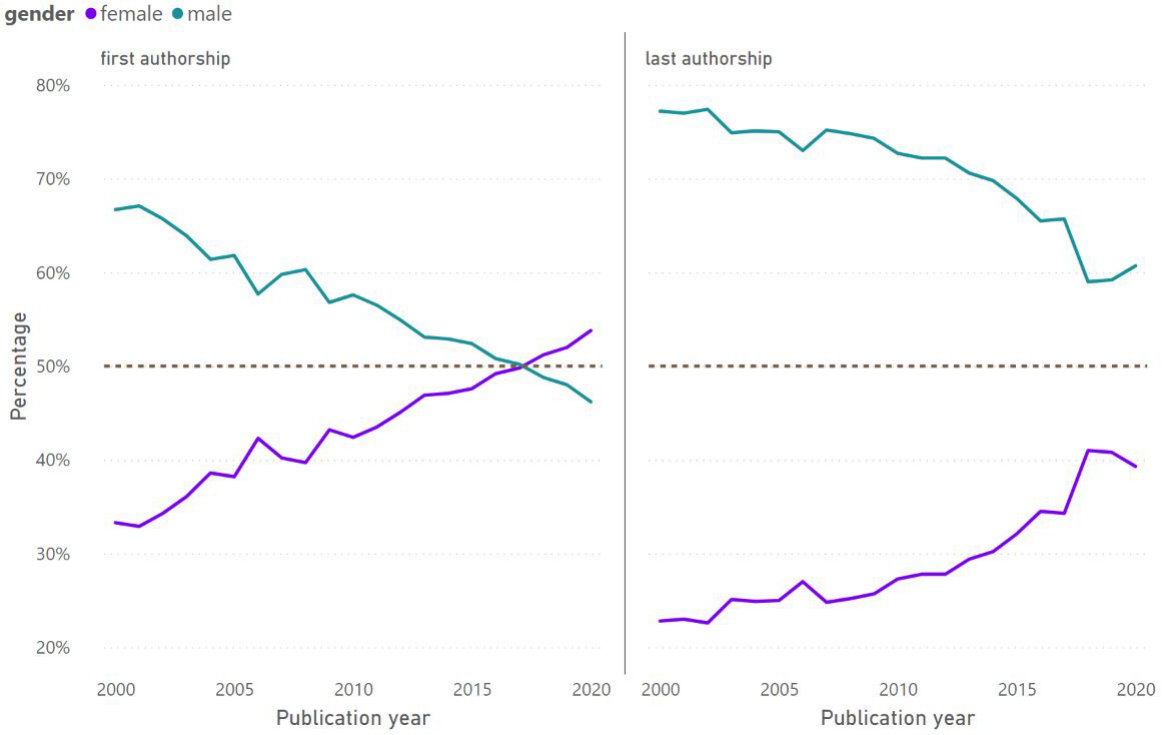
Gender distribution in first and last authorships between the years 2000 and 2020.

Figure 3 examines the trend in gender disparities among first authorships, representing authors in the early stages of their careers, and last authorships, representing authors later in their careers. Female first authorships surpassed male first authorships in 2018 and continued increasing. At the end of the study timeframe, the gender ratio was still 1.5 for last authorships. However, the increase in female representation in the field has come about unequally among first and last authors. The pace of change in last authorships has been slower than for first authorships (slope of 0.9987 vs. 0.8621). Furthermore, last authorships have seen a slight regression for female authors, particularly in the year 2020.

### Analysis of ARUK’s grant application data

ARUK awards funds in various grant schemes and funding agreements that are based on open calls (e.g., Major Projects, Fellowships) and closed calls (i.e., assigned to a principal investigator based on an existing partnership, agreement, or strategic initiative). We focused our analysis only on competitive grants. For the purposes of this analysis, we divided ARUK’s grants into three categories: Fellowships, Major Projects, and Other Senior Projects. Fellowships comprise all the early career grants; Major Projects are the highest-value grants and usually awarded to researchers established in their fields; and Other Senior Projects comprise all our other grants for senior researchers, including scientists with a tenure position or a long-term record of accomplishment.

Overall, women applied to 38% of our competitive grants, received 37% of the grants and 37% of the funding awarded.

The proportion of applications from female researchers decreases from 59% in Fellowships to 35% and 39% in Major Projects and Other Senior Projects, respectively ( Table 6). Chi-squared analysis showed a significant relationship between gender of the applicant and types of grants they apply to (p-value <0.001).

**Table 6.** Gender distribution of applications received by type of project.

	Total	Female		Male	
Applications received	n	n	%	n	%
Fellowships	352	207	58.8	145	41.2
Major Projects	361	126	34.6	236	65.4
Other Senior Projects	1237	478	38.6	759	61.4

**Figure 4.**
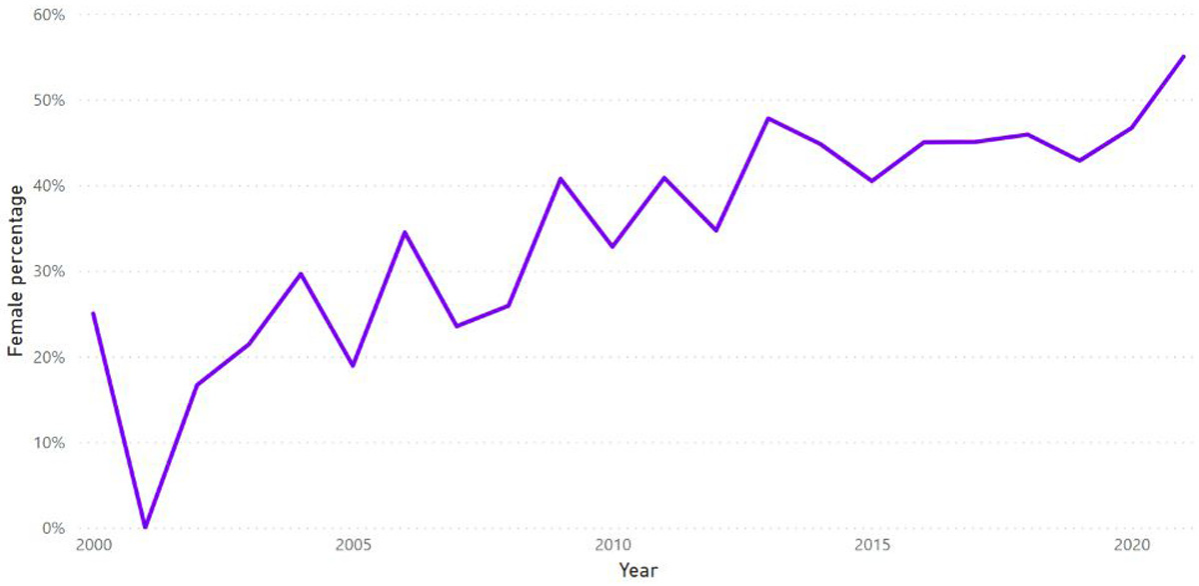
Percentage of applications from female researchers received by ARUK between the years 2000 and 2021.

The trend of the gender of applicants over time was examined. There is a clear upward trajectory in the percentage of female applicants to ARUK grants ( Figure 4).

We examined the relationship between gender, type of grant and outcome ( Table 7). The estimated Mantel-Haenszel (MH) summary (crude) odds ratio was 1.30 (95% CI 1.05 to 1.59, p-value = 0.013), which indicates that on average being male is strongly associated with 1.30 higher odds of having a successful application compared to being female. Among Fellowship projects, there was no evidence for a difference of the outcome when comparing male and female applicants (p-value = 0.5732). The odds of male applicants being funded were 1.91 (p-value 0.016) and 1.31 (p-value = 0.033) when they apply to Major Projects and Other Senior Projects, respectively.

**Table 7.** Mantel-Haenszel odds ratios (outcome and gender), controlling for type of grant.

Stratified by Type of Grant	MH OR (95% CI)	P-value
Fellowships	0.86 (0.52, 1.43)	0.573
Major Projects	1.91 (1.11, 3.29)	0.016
Other Senior Projects	1.31 (1.02, 1.69)	0.033
Average	1.30 (1.05, 1.59)	0.013

Multivariate analysis showed that male applicants had 1.29 times the odds of being funded compared to females, after adjusting for the type of project (adjusted OR 1.29, 95% CI: 1.05–1.59, p-value = 0.004) ( Table 8). This means that the odds of being funded increased by 29% when the gender of the applicant was male, after adjusting for the type of project.

**Table 8.** Univariate and multivariate analysis of ARUK application outcome.

		Univariate analysis		Multivariate analysis	
		OR (95% CI)	P-value	OR (95% CI)	P-value
Gender					
	Female	1		1	
	Male	1.35 (1.10, 1.65)	0.004	1.29 (1.05, 1.59)	0.013
Type of Project					
	Fellowship	1		1	
	Major Projects	1.26 (0.89, 1.77)	0.181	1.18 (0.83, 1.67)	0.335
	Other Senior Projects	1.66 (1.26, 2.18)	<0.001	1.58 (1.19, 2.08)	0.001
Intercept		---		0.29 (0.22, 0.38)	<0.001

**Figure 5.**
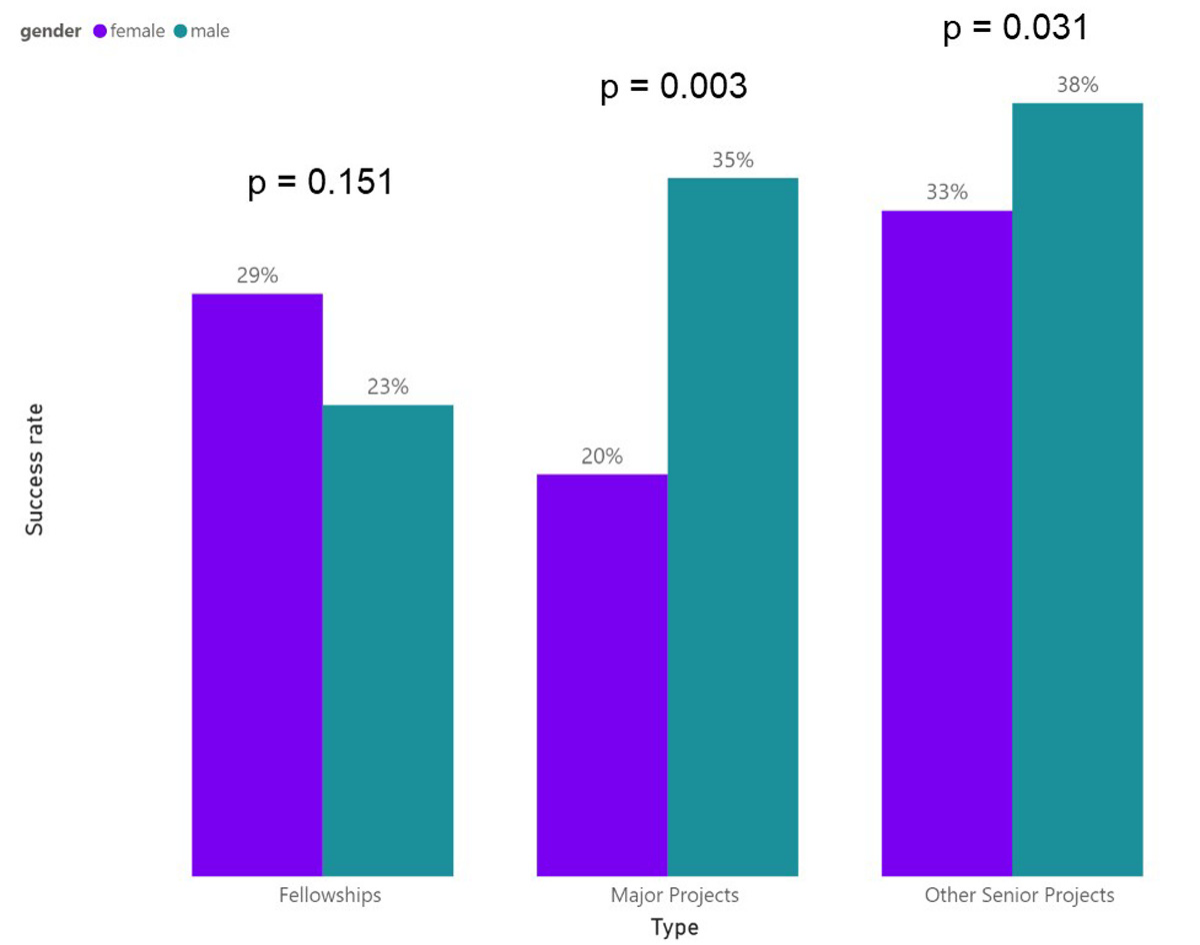
Success rates to each grant type by male and female researchers.

Another way of examining this data is to calculate the success rates of the different grant schemes. Success rate is the ratio between successful applications and the total number of applications. We observed in the ARUK data that men and women presented different success rates for the different grant schemes ( Figure 5). While women appear to have higher success rates when they apply to Fellowships (29% for female researchers vs. 23% for male researchers, p-value = 0.151), they are consistently less successful in obtaining funding via senior grants (Major Projects: 20% females vs. 35% males, p-value = 0.003; Other Senior Grants: 33% females vs. 38% males, p-value = 0.031)

ARUK grants are evaluated in a two-stage process. First, applications are reviewed by an international group of peer reviewers, where reviewers score grants on a 1 to 5 scale, with 1 being the best possible score and 5 the worst. Second, the grants are evaluated by ARUK’s Grant Review Board (GRB), a panel of senior dementia researchers based in the UK.

**Figure 6.**
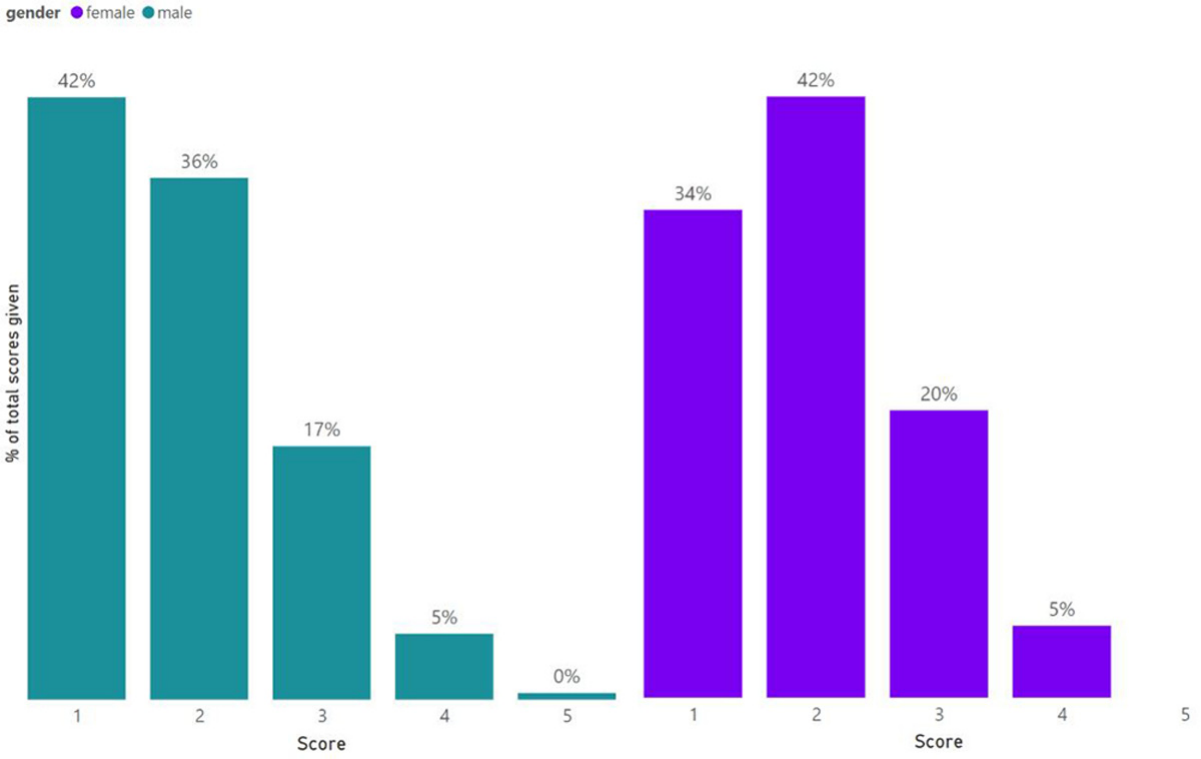
Score distribution of ARUK grant applications by gender of the reviewer.

Of the 1,384 scoring instances where the gender of the peer-reviewer was known, 70% of them were conducted by male reviewers. Scores from male reviewers were on average lower (i.e., better scores) than those from female reviewers (p-value = 0.014), as seen in the distribution of scores by male and female reviewers in Figure 6.

The results above are the averages across all types of grants. We examined the grants with mixed-gendered panels of reviewers to see how the reviewers’ individual scores compared to the average score of the grant. This was categorized as scoring “better than the average score”, “worse than the average score”, or the “same as the average score” of the grant ( Table 9). Male reviewers gave better scores than women more often (41% vs. 37%) when they evaluated the same grant. However, these gender differences among reviewers were not statistically significant (p-value = 0.378).

**Table 9.** Percentage of female and male reviewers that score grants better, worse or the same as the score average.

	Female reviewer	Male reviewer
Give better score than average	37%	41%
Give same as average score	28%	29%
Give worse score than average	35%	31%

**Figure 7.**
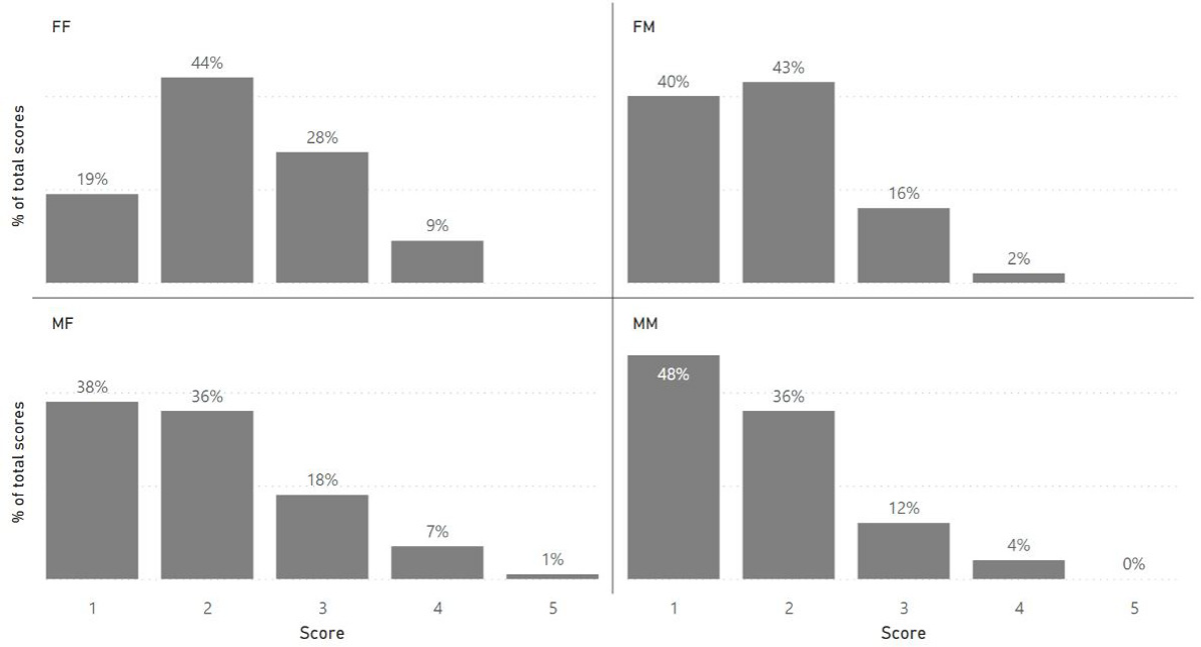
Score distribution of ARUK grant applications by the relationship gender of the reviewer and gender of the applicant.

Finally, we investigated how the interaction of the gender of the applicant with the gender of the reviewer may influence the scoring. Grants were divided into the following categories: MM (male reviewing a grant with a male lead applicant), FF (female reviewing a grant with a female lead applicant), MF (male reviewing a grant with a female lead applicant), and FM (female reviewing a grant with a male lead applicant). We analysed the score distributions for all grant schemes, and the most noteworthy differences were found for the Major Project schemes. For these grants, pairwise comparisons showed that men score men (MM) better than women score women (FF) (p-value <0.001); men score men (MM) better than men score women (MF) (p-value = 0.028); men score women (MF) better than women score women (FF) (p-value = 0.028); and women score men (FM) better than women score women (FF) (p-value = 0.002) ( Figure 7).

At Grant Review Board stage, female members of the Board gave on average better scores than male members (p value < 0.001), which is the opposite of what was seen in the previous review stage. However, the overall difference in score based on reviewer gender was smaller in relative terms ( Table 10). Scores at the Grant Review Board stage are also higher (i.e., considered worse) and have a narrower spread than those from the peer-review stage.

**Table 10.** Average scores and relative gender differences at peer-review and Grant Review Board stage.

Review stage	Overall average	Female reviewer average score	Male reviewer average score	Relative Difference
Peer review	1.89	1.96	1.86	5.3%
Grant Review Board	2.51	2.45	2.56	-4.4%

## Discussion

This study provides a complete view of the academic dementia research career pipeline during the last two decades, with a focus on data collected by ARUK on its grant schemes. The analysis leads to several insights:

### Women comprise the majority of the dementia research field today

Attendance at traditional in-person conferences did not used to mirror the exact demographics of a research field. Researchers working in organisations or countries with fewer resources, and those with caring responsibilities, were less likely to attend such events. The shift to online conferences since the beginning of the COVID-19 pandemic has allowed for broader audiences to attend these events, better reflecting the true makeup of the research population ^32^. In overall terms, today the majority of the dementia research field is female, though there are marked gender differences based on career stage.

### Women publish fewer papers on average

We have seen that the male:female gender ratio is 1.15 for authors, 1.5 for authorships, and it goes up to 2.25 for last authorships. We show that the overall difference in “productivity” is significant only at senior stages in academics’ careers, and we postulate that it is due to the bigger number of male researchers in senior positions. Scientific productivity along a two-decade span is intertwined with career longevity, so authors who progress further in their academic careers will almost inevitably publish more ^33^. In early career stages, the gender disparities in overall productivity levels are small. It is only when we look at those who have become prolific authors that we see stark differences between men and women. Given the underrepresentation of women in last authorships (which is a proxy for career seniority), it is not surprising to find that most of the difference in authorships between male and female researchers comes from authors who are highly prolific.

### The gender gap is closing, but unequally

Our results agree with other analyses that show that the gender gap in academic research is closing. However, we demonstrate that it is doing so at different rates for junior and senior researchers. The rate at which junior female researchers are joining the field has been higher than the rate at which female researchers have transitioned into senior positions.

This difference in growth rate further indicates that female researchers do not progress to positions of seniority at the same rate as men. Given the lag time in career progression from an ECR position to a senior academic position, it is expected that female last authorships will continue to increase over the coming years.

It is noteworthy to mention the marked decrease in female last authorships in the year 2020, hinting at the unequal effects of the COVID-19 pandemic in researchers’ careers. This could be due to the unbalanced distribution of household work and caring responsibilities between men and women. It is worth considering this in any type of future “productivity” analysis that may influence funding or career progression decisions.

### Applicant gender plays a role in the application and success rates of each grant scheme

There is an upward trend in applications from women to ARUK’s grant schemes. The percentage of women applying each year can vary due to the fact that ARUK opens different grant schemes for funding in different years, and each scheme has a different gender composition influenced by the career stage at which they are aimed. Women apply more often to early career calls (Fellowships) than senior grant calls (Major Projects and Other Senior Projects).

The analyses showed that after adjusting for the applicants’ advancement in their careers, there was evidence of male applicants having higher odds of having successful applications. Stratified data showed there was no effect of gender for Fellowships, but in Major Projects and Other Senior Projects male applicants were more likely to be awarded a grant.

The results show female applicants having worse success rates in more senior career project applications. As we saw in the bibliometric data in the previous section, this points towards the dementia research field losing female researchers along the career path to senior positions or female researchers remaining stagnant in their careers.

### Grant scoring is affected by the gender of the reviewer

The results suggest that male and female reviewers may apply ARUK evaluation criteria differently, which leads to score differences based on the gender of the reviewers. Men appear to give better scores than women on average, although the differences become non-significant when we analyse whether men and women score the same grant differently. The interaction between the gender of the reviewer and the gender of the applicant seems to play an important role particularly in Major Projects. The pairwise comparison between the different categories (MM, MF, FF and FM) showed that both male and female reviewers score male applicants better than they do female applicants. This would help explain the significant difference in success rates between male and female application to Major Projects (35% vs. 20%, p-value = 0.003).

Finally, the differences in average scores between the peer-review and the GRB stage are noticeable, with GRB scores being considerably worse on average. Scoring differences based on the gender of the reviewer at the GRB stage are also relatively smaller than at peer-review stage. We hypothesise that this is because two members of the Board are required to present their assigned grant applications to all other members and disclose publicly how they are scoring that grant. This may provide a benchmark from which the rest of the members of the Board are unlikely to deviate significantly.

### Study limitations

The findings of this study have to be interpreted with some limitations in mind. First of all, the gender of researchers, grant applicants, and reviewers were inferred based on the authors’ first names. This was conducted using a validated tool and the inferred gendered was manually checked for a sub-set of the sample by assessing researchers’ pictures to decrease potential errors. However, this binary classification is limiting and ideally researchers would be able to self-identify.

Second, due to the limitations on the confidence on the gender assignment based on first names, this study was limited to the top 10 Western countries by number of dementia research publications, and authors without typical Western names were disproportionally removed from the dataset. This means that the findings are not generalizable to all worldwide dementia researchers.

Finally, all funding data was specific to ARUK grant schemes and review processes.

## Conclusions

A number of reasons have been suggested in the literature to help explain some of the gender differences we have observed in this analysis. Based on these conclusions, ARUK has implemented or is working to implement interventions to address these disparities.

It is possible that grants with different evaluation criteria may inadvertently lead to outcome differences influenced by gender ^18^. ARUK reviewers are instructed to score the early career grants based on a detailed competency framework ( https://www.alzheimersresearchuk.org/wp-content/uploads/2015/02/Early-Career-Researcher-Framework-Nov18.pdf), while the guidelines for senior grants are broader and less well defined. This can lead to reviewers applying different criteria, which might be influenced by personal (unconscious or conscious) biases. We are working to provide our reviewers with better-defined evaluation criteria in all future grant rounds. 

Past productivity of male and female academics is one of the main evaluation criteria that can influence the outcome of an application. First, ARUK questions whether the current definition of academic “productivity”, which seems to systematically benefit certain groups of researchers, is the right one. There are other types of academic outputs, such as teaching, mentoring, and engagement activities, all of which benefit the research community. By expanding the types of academic outputs when evaluating career tracks, we can better recognise the work of all researchers. Moreover, as a medical research charity funder our main consideration must be the potential future impact of the research on the patient population, and traditional definitions of academic impact do not necessarily align with these considerations. Second, the literature suggests that reviewers may inadvertently become biased when they assess candidates’ applications. For example, reviewers may be swayed by indicators of “prestige” in CVs such as lists of papers with first authorships, publications in high impact factor journals, or lists of past grants ( https://sfdora.org/resource/rethinking-research-assessment-unintended-cognitive-and-systems-biases/). To address this, ARUK is piloting the use of narrative CVs, in which applicants are asked to describe their specific contributions in a limited number of outputs of their choosing. The authors of this manuscript are also listed alphabetically – this contravenes the conventional model of authorship that facilitates biased assessments of authors’ contributions and gives way to some of the disparities discussed in this analysis.

Moreover, ARUK is striving to offer more support to early career researchers to minimise the effect of external factors on their career progression. The new ECR programme ( https://www.alzheimersresearchuk.org/research/for-researchers/ecr/) offers mentoring and training opportunities, which has a focus on helping researchers who face additional barriers in the early stages of their careers make the transition into senior positions. ARUK also opened new bridge funding opportunities.

ARUK recognises that all these measures do not address perhaps the tallest barrier that women face in academic research: that they are often seen as less capable researchers than their male peers ^34^. Studies trying to address biases in the peer-review process that lead to gender and ethnic disparities have compared the use of single-blind (i.e., the reviewer knows the identity of the author, but not the other way around) vs. double-blind (i.e., the identity of both reviewer and author are unknown to each other). While evidence is mixed, some of these studies have found that double-blind improves the quality of the review process by lowering the scores given to prestigious authors and universities ^20, 35^; single-blind hinders the arrival of newcomers to a research field ^36^; and double-blind increases representation of female authors ^21^. ARUK will pilot the blinding of applications to the reviewers of one of our grant calls, and based on the results we will consider expanding the initiative to all grants in the near future.

Several of the issues identified in this paper are systemic and not unique to a single funder. ARUK will work in collaboration with other funders and share our learnings during this process in order to identify and dismantle barriers for talented women in other fields of academic research. A first step in the right direction for other funding institutions is to analyse and publicly disclose similar analyses of their research grants. Only by all funders systematically gathering data on protected characteristics and monitoring their internal funding processes can we hope to uncover and address the biases that exist in academic research.

Finally, this analysis and discussion was limited to gender, but ARUK will conduct similar analyses for other protected characteristics and aim to develop innovative ways to support and retain under-represented researchers in the field of dementia research.

## Data availability

### Underlying data

Zenodo: Underlying data for ‘The dementia research career pipeline: Gender disparities in publication authorships and grant funding outcomes at different career stages’. ‘Dementia career pipeline gender analysis’. https://doi.org/10.5281/zenodo.6603650 ^30^.

This project contains the following underlying data:

- ARUK Grant Applications and Outcomes – manual gender check.xlsx
- ARUK Grant Review Board scoring – manual gender check.xlsx
- ARUK Peer Review scoring – manual gender check.xlsx
- Dimensions gender analysis.R
- Visualisations of ARUK data.pbix
- Visualisations of Dimensions data.pbix
- ReadMe.md

Data are available under the terms of the Creative Commons Zero “No rights reserved” data waiver (CC0 1.0 Public domain dedication).

**Zenodo**: Underlying data for ‘The dementia research career pipeline: Gender disparities in publication authorships and grant funding outcomes at different career stages’. ‘Dimensions data’. https://doi.org/10.5281/zenodo.6535739 ^31^.

This project contains the following underlying data:

- Dimensions-Publication-2021-10-14_10-46-47.csv
- Dimensions-Publication-2021-10-14_10-47-34.csv
- Dimensions-Publication-2021-10-14_10-48-08.csv
- Dimensions-Publication-2021-10-14_10-48-26.csv

Data are available under the terms of the Creative Commons Attribution 4.0 International license (CC-BY 4.0)

## Software availability

- Source code available from: https://github.com/jorgoma/gender-analysis-paper
- Archived source code at time of publication: https://doi.org/10.5281/zenodo.6603650 ^30^.
- Creative Commons Zero "No rights reserved" data waiver (CC0 1.0 Public domain dedication).
