# Supplementary material for: The dementia research career pipeline: Gender disparities in publication authorships and grant funding outcomes at different career stages
Source: AMRC Open Res. 2022 Aug 10;4:18. [Version 1] doi: 10.12688/amrcopenres.13072.1 (PMC11064979; doi:10.12688/amrcopenres.13072.1)
Supplement: Peer review history

# Reviewer report: Reviewer response for version 1

**DOI:** 10.21956/amrcopenres.14150.r27263

**Published:** 2023-07-19

**Authors:** C. Elizabeth Shaaban

**Affiliations:** 1 University of Pittsburg, Pennsylvania, USA

Thank you for inviting me to review this manuscript assessing for gender disparities in dementia research based on conference attendance; paper first-, last-, and co-authorships; and grants reviewed and funded through Alzheimer’s Research UK. The study presents descriptive and some regression-based modeling to evaluate these relationships. This topic will be of interest both in the dementia research field as well as more broadly. The evaluation of gender disparities in numerous measures is a key strength of this well written and clear paper.

 I offer the following comments to strengthen the manuscript, and I hope they are helpful for revising. They are divided into major and minor comments. On any question where I have indicated “partly”, more detail is included in my specific comments below.

 Sincerely,

 C. Elizabeth Shaaban

 University of Pittsburgh

 ######

 MAJOR

 ######

 1. Throughout the paper, “male”, “female”, “men”, and “women”, are used interchangeably. This paper is about gender, and thus should use terms referring to gender rather than sex; e.g., use “women” and “men” not “male” and “female” which refers to sex assigned at birth and related “sex” constructs rather than “gender”.

 METHODS

 2. What is Dimensions? Why was it selected over PubMed? Did this limit publications to those funded by the funders listed in Table 2 vs also including other funders like government funders? If so, why were government funders excluded? More information about this methodologic choice should be provided.

 ######

 MINOR

 ######

 METHODS

 1. In the methods, the authors state, “To double-check the accuracy of the API, we manually searched 468 ARUK grant awardees.” What happened when this was done? Please add this to the results section.

 2. More information about how gender was determined for each piece of the analysis is needed. How was the gender of AAIC attendees determined? I would imagine it was by self-report. The specific question and answer choices be listed? I think the gender for the authorship was using the gender api. Can they authors say if the gender used for the ARUK analysis was based on picture identification, the gender api, or self-report? If self-report, what was the wording of the question and answer choices?

 3. What years was the grant application and grant scoring data pulled from?

 4. The authors state “Using the ARUK grant application data, the effect of the gender of the applicant on the outcome of the funding application was examined using descriptive analysis “ Because the analytic approach is overall more descriptive and not designed to be a causal analysis, I would change this to say the *association* of gender with the funding outcome was examined.

 5. The word multivariate is used in multiple places in the manuscript (e.g., “To control for confounding factors and to assess for potential effect modification, the data were assessed by stratification using Mantel-Haenszel and multivariate regression. “), but the correct term is multivariable.

 6. Can the authors clarify in the methods if they tested effect modification via interaction terms in the regression modeling? If so, this could also be made clearer in the results sections.

 7. For the AAIC Conference data, I would suggest adding 2022 (and very soon 2023). Adding these two years helps to give a better sense of what is happening since COVID rather than just 2020 and 2021.

 RESULTS

 8. The terminology of “authors” vs. “authorships” is confusing. Is there another way to refer to this or a way to put a legend with a reminder of this in the tables and figures that use this terminology?

 9. Table 2 needs to spell out abbreviations like HRCS, RCDC in the table or needs a legend spelling them out.

 10. The authors state: “Another way of thinking about this ratio discrepancy between authorships and authors is in terms of the average number of publications each researcher produces: 3.1 publications co-authored by female researchers and 4.2 co-authored by male researchers, a 35% difference.” This should be tested statistically. Was this statistically different? Please add to the results and make sure the methods for this testing are included in the methods section.

 11. Table 5 is redundant to Figure 2. Consider adding data labels with the %s at the end of the bars in Figure 2 and then it would be possible to remove Table 5.

 12. The authors report authorships slopes for first and last-authored papers (slope of 0.9987 vs. 0.8621). Did the authors test to see if these slopes are different? The authorship type (first-, co-, or last-authored)*year interaction should be tested to see if this is significant. Then the stratified slope for first-authored and slope for last-authored papers should be reported after that. It is also helpful for the reader if the authors interpret what the slope means. For each year that passes, women’s 1 ^st^ authorship increased 1% while women’s last authorship increased by 0.86%. Please make sure these analyses are also described in the methods section.

 13. Is figure 7 for all types of grants or only the Major Project schemes? This should be added to the legend.

 DISCUSSION

 14. Please add to the limitations that the analysis was complete case analysis and did not account for missing data.

 15. The authors comment that “Furthermore, last authorships have seen a slight regression for female authors, particularly in the year 2020.” Does this suggest that this regression may have been happening prior to the pandemic then? Any ideas on the literature why that may be?

 16. It’s worth pointing out in the discussion that a number of statistical tests were performed, and p-values were not adjusted for multiple comparisons. Readers should keep this in mind when considering the results.

 17. The authors discuss that the majority of dementia researchers today are women. Is there a citation for this or a comment on what within their own data supports this?

 OTHER

 18. Declaration of Interest—I suggest reporting the authors’ positions at ARUK just to be completely transparent.

Is the work clearly and accurately presented and does it cite the current literature?

Yes

If applicable, is the statistical analysis and its interpretation appropriate?

Partly

Are all the source data underlying the results available to ensure full reproducibility?

Yes

Is the study design appropriate and is the work technically sound?

Yes

Are the conclusions drawn adequately supported by the results?

Yes

Are sufficient details of methods and analysis provided to allow replication by others?

Partly

Reviewer Expertise:

Epidemiology; Alzheimer's disease and related dementias (ADRD); sex and gender in ADRD; career development of dementia researchers

I confirm that I have read this submission and believe that I have an appropriate level of expertise to confirm that it is of an acceptable scientific standard, however I have significant reservations, as outlined above.

# Reviewer report: Reviewer response for version 1

**DOI:** 10.21956/amrcopenres.14150.r27257

**Published:** 2023-07-19

**Authors:** Millicent Liani

**Affiliations:** 1 International Institute of Tropical Agriculture, Ibadan, Oyo, Nigeria

Thank you for the opportunity to review this article. This is a novel article that is nicely written article that unravels gender disparities in publication authorships and grant funding outcomes at different career stages in the dementia research career pipeline. The analysis is mainly based on the Alzheimer’s Research UK’s (ARUK) data. The authors suggest interventions that ARUK can implement in its application and evaluation process to address gender disparities. I have minor comments and suggestions that should be addressed to help enrich the article:

1. Most of the cited content is from global north. This could be specified in the title if the study focused on career pipeline in the North. The authors should also provide citation to the first two paragraphs in introduction….is it a global trend or specific to UK?
2. Throughout the paper, the authors use the framing ‘men and women’ as opposed to the vice versa. We all know that compared to men, women continue to suffer from historically marginalization and are considered vulnerable and disadvantaged. Indeed, within the discourse of gender studies, such framing of ‘men and women’ is a rhetoric language that contributes to further subjugation of women. The authors should address these changes in the article.
3. The authors note that they have observed that male and female reviewers apply evaluation criteria differently, with men giving better scores than women on average. This is a unique finding for which they should provide a detailed discussion on this supported by the literature.
4. There is repetition of the content in the result and discussion section. This could be addressed by merging the ‘results and discussion’ sections as one section where the authors highlight the findings as they discuss them. The discussion should be enriched with more empirical evidence. So far only two citations are provided. For instance, the UK Athena Swan charter encourages and recognises commitment to advancing the careers of women in STEMM HEIs, currently used across the globe to address gender equality more broadly, and not just barriers to progression that affect women. How does this apply to this study? There is also a global debate on whether provision of funding to researchers at different career stages should be based on the scientific excellence model or scientific merit and equity criteria, a matter that needs to be addressed with urgency – see Chataway et al. (2019). Science granting councils in Sub-Saharan Africa: Trends and tensions. *Science and Public Policy, 46(4), 620–631*.

I look forward to seeing this important piece of work indexed!

Is the work clearly and accurately presented and does it cite the current literature?

Partly

If applicable, is the statistical analysis and its interpretation appropriate?

I cannot comment. A qualified statistician is required.

Are all the source data underlying the results available to ensure full reproducibility?

Yes

Is the study design appropriate and is the work technically sound?

Yes

Are the conclusions drawn adequately supported by the results?

Yes

Are sufficient details of methods and analysis provided to allow replication by others?

Yes

Reviewer Expertise:

Equitable scientific research career pathways; research capacity strengthening; intersectionality, systems of career progression in science research; gender responsive and transformative approaches.

I confirm that I have read this submission and believe that I have an appropriate level of expertise to confirm that it is of an acceptable scientific standard, however I have significant reservations, as outlined above.

## References

[1] : Science granting councils in Sub-Saharan Africa: Trends and tensions. *Science and Public Policy* .2019;46(4) : 10.1093/scipol/scz007 620-631 10.1093/scipol/scz007

# Reviewer report: Reviewer response for version 1

**DOI:** 10.21956/amrcopenres.14150.r26979

**Published:** 2022-08-22

**Authors:** Tara Spires-Jones

**Affiliations:** 1 Centre for Discovery Brain Sciences and UK Dementia Research Institute, University of Edinburgh, Edinburgh, UK

Andreou et al. present an analysis of gender disparities in academic research with a focus on dementia researchers. Overall this is a clear, well-written, and important article that will be of wide interest to the field.

 A few comments for improvement:

- It would be good to clarify from the start (in the abstract and introduction) that this analysis focuses on dementia research.  This is important because some statements like that of authorship order are not correct for all academic fields. For example, in some fields, the convention is to list all author alphabetically.
- Table 2 is confusing - what does the “best grant coverage” mean?  It is not clear what the percentages in the table represent.
- It is surprising that data on attendance at the Alzheimer’s Research UK conference was not included as this parallels the grants analysed.
- In figure 2, the difference between “authors” and “authorships” is not clear.
- It would be very interesting to know about the non-competitive funded grants given by Alzheimer’s Research UK as there could also be gender disparities there that are more “hidden” as these do not come from open calls.

Is the work clearly and accurately presented and does it cite the current literature?

Yes

If applicable, is the statistical analysis and its interpretation appropriate?

Yes

Are all the source data underlying the results available to ensure full reproducibility?

Yes

Is the study design appropriate and is the work technically sound?

Yes

Are the conclusions drawn adequately supported by the results?

Yes

Are sufficient details of methods and analysis provided to allow replication by others?

Yes

Reviewer Expertise:

Neurodegeneration, synapse loss, Alzheimer's disease

I confirm that I have read this submission and believe that I have an appropriate level of expertise to confirm that it is of an acceptable scientific standard.
